# Behavior-analytic intervention for women with fibromyalgia and insomnia: a single subject design

**DOI:** 10.1186/s41155-020-00169-2

**Published:** 2021-02-08

**Authors:** Luziane de Fátima Kirchner, Maria de Jesus Dutra dos Reis

**Affiliations:** 1 Federal Univerisity of São Carlos–UFSCar, KM 235 Washington Luis, São Carlos and São Paulo, 13565-905 Brazil; 2 Dom Bosco Catholic University–UCDB, 6000 Tamandaré Avenue, Jardim Seminário, Campo Grande and Mato Grosso do Sul, 79117-900 Brazil; 3 University of São Paulo, São Paulo, Brazil

**Keywords:** Fibromyalgia, Insomnia, Behavioral intervention

## Abstract

This study evaluated the effects in the pain and sleep, and the clinic significance after an analytic-behavioral intervention to manage the condition of the physical and interpersonal environment related to pain. Four women with fibromyalgia and insomnia participated in a study with intervention withdrawal multiple baseline design and initial, intermediate, final, and follow-up assessments. Self-report instruments were used to assess pain intensity and disability, sleep quality, and insomnia severity, besides the actigraphy. Data showed that the intervention (20 sessions) was effective in reducing the sleep and pain problems in all participants by shifting two participants from clinical to non-clinical status in sleep indicators. The gains were maintained or increased in follow-up measures. However, the results should take into consideration the clinical condition and other variables that may have individually impacted the results.

## Introduction

According to the International Association for the Study of Pain, pain would be an unpleasant sensory and emotional experience associated with or related to real or potential tissue damage (International Association for the Study of Pain - IASP, 1994). The painful sensation when prolonged exposes its carriers to conditions of severe stress, affecting the physical and mental functioning. Continued living with the painful process can lead the individual to a condition called painful disorder (Linares, Pérez, Pérez, Lima, & Castaño, 2005).

In the Diagnostic and Statistical Manual of Mental Disorders, DSM-V-TR (2014), the criteria for identifying painful disorders were included in the Somatic Symptom Disorders and Related Disorders section, cited as the diagnostic category: (A) one or more somatic symptoms that are particularly stressful or result in significant disruption or deficit in daily functioning; (B) the excessive presence of behaviors, feelings, or thoughts related to pain and persistence of the symptoms for a period longer than 6 months. Damages to social, occupational, or other important areas have been recognized as an ordinarily present element of chronic pain (Melzack & Wall, 1965; Thieme & Turk, 2012). A chronic painful syndrome associated with widespread impact on daily routine and social functioning has been fibromyalgia (FM). This usually covers the chronic presence of painful and diffuse processes, identified by the clinical finding of painful sensitivity in 11 of 18 tender points (Wolfe et al., 2010). This picture shows significant psychiatric comorbidity with anxiety disorders, depression (Thieme, Flor, & Turk, 2006), and sleep disorders, with a higher prevalence of insomnia (Prados & Miró, 2012).

For Fordyce, behaviors like moving slowly, complaints about the pain and self-medicating are the results of a long-term learning and they were reinforced in the environment. On the other hand, the adaptive behaviors related to health care were extinguished (Fordyce, 1976; Main, Keefe, Jensen, Vlaeyen, & Vowles, 2014).

The World Health Organization (Linton, 1993), in a document published more than 20 years ago, already pointed to some behavioral strategies recognized as effective for the intervention of chronic pain, particularly back pain, as follows: relaxation techniques (e.g., progressive, autogenous, biofeedback, among others); operant behavior change techniques (e.g., educational strategies, increased physical activity, reduced medication intake, among others); cognitive strategies (e.g., distraction techniques); social skills training (in particular assertiveness); and coping strategies. Over the years, research has corroborated the effectiveness of these techniques for a variety of chronic pains, including fibromyalgia (Nicassio et al., 1997; Thieme, Gromnica-Ihle, & Flor, 2003).

Functionally such strategies would consist in identifying immediate or delayed reinforcers and pain contingents, seeking to intervene in the establishment of new contingencies aimed at increasing adaptive behaviors (Fordyce, 1976; Main et al., 2014). Usually, functional analysis is conducted and relate to each individual’s pain behaviors, seeking to (1) establish repertoire that reduces the physiological effects of pain and anxiety through relaxation training; (2) develop and strengthen behaviors that re-establish a richer and more adequate functioning in physical and occupational activities; (3) develop more effective responses in establishing more positive social interactions. These strategies are grounded in the literature and scientifically tested (Main et al., 2014; Sanders, 2006), with particular emphasis on those studies that conducted group intervention analyzes (Morley, Eccleston, & Williams, 1999; Thieme et al., 2006).

However, the clinical variability of conditions in this population, together with the contingencies established in the relationship between behavior and environment, may have a different impact on treatment outcomes and are justifications for conducting research with single-case research design, obtaining analyzes of the individual as their own control (Gast, 2010; Kazdin, 1982). As pointed out by Morley, Linton, and Vlaeyen (2015), we need to take into account the methodological features that enable the visualization of more accurate functional relationships between interventions and outcomes. Single-case design can be one of these alternatives by providing continuous feedback on treatment progress for each participant.

In a search in the databases of Portal Capes (Index Psi, Pepsic, Scielo, and BVS), using the keywords “single-case AND pain AND behavior analysis,” it was possible to verify that only a few behavioral intervention studies using single-case designs were found for people with chronic pain, including FM (Äsenlöf, Denison, & Lindberg, 2005; Gómez-Pérez, García-Palacios, Castilla, Zaragozá, & Suso-Ribera, 2020; Lundervold, Talley, & Buermann, 2006; Lundervold, Talley, & Buermann, 2008). These studies measured, besides the intensity and inability of pain, variables such as anxiety and depression (Gómez-Pérez et al., 2020; Lundervold et al., 2006; Lundervold et al., 2008) and coping with pain strategies (Äsenlöf et al., 2005). Until the present moment, none of the studies proposed the use of sleep and insomnia measures.

The literature indicates an important correlation between intensity and inability of pain and changes in the macro and microstructure of sleep in people with FM (Bennett, Clark, Campbell, & Burckhardt, 1992; Keskindag & Karaaziz, 2017; Rizzi et al., 2016; Roizenblatt, Moldofsky, Benedito-Silva, & Tufik, 2001). Bennett et al. (1992) observed that there was an increase in pain, fatigue, and pro-inflammatory cytokines in female patients with FM who had slow-wave sleep deprivation. Roizenblatt et al. (2001) also showed that people with severe pain had more difficulties to sleep, characterized by the difficult to start the sleep and smaller amount of sleep throughout the night. Keskindag and Karaaziz (2017) still suggested that people with disabilities because of the pain can spend more time resting and in isolation, what can compromise the quality and the pattern of sleep throughout the night. According to Finan (2018), the mechanism of action between pain and sleep problems are not clear yet, but it is known that the presence of both conditions can establish a cycle in the severity of the symptoms, if one of them is not treated. Interventions that assess the impact on these variables, pain and sleep, can add important theoretical value, by identifying how they interact together, and clinical value, in order to show how they interfere in the effectiveness of the results.

Some studies of analytical and cognitive behavioral intervention for insomnia in people with FM, show effects in the quality and sleep pattern, as well as in functional capacity related to pain (Edinger, Wohlgemuth, Krystal, & Rice, 2005; Miró et al., 2011), what brought up the following question: interventions of the analytic-behavioral approach directed to the management of chronic pain can produce changes in sleep if they are also effective in pain? This study focused on evaluating the effects of an intervention analytic-behavioral involving two components about pain indicators and sleep in women with FM and insomnia, to know (1) management of physical environment conditions; (2) managing interpersonal relationships. A direct measure (actigraphy) was used in this study to evaluate the effects of the treatment in the sleep (Fekedulegn et al., 2020). In addition, clinical significance was assessed using the JT method (Jacobson & Truax, 1999), which identifies whether the results attributed to intervention, based on scores of the instruments applied in initial and final evaluations, move between the representative ranges clinical and non-clinical.

## Method

### Participants

There are four women taken part of this study and they show medical diagnoses of fibromyalgia, diagnostic symptoms according to Wolfe et al. (2010), and insomnia disorder identified according to the criteria of the Diagnostic and Statistical Manual of Mental Disorders, DSM-V-TR (2014) and actigraphy data (Octagonal Basic Motionlogger®, Ambulatory monitoring; Actwath-64®, Phillips Respironics). Besides those inclusion criteria, the participants could not show other sleep disturbance identified by a health professional (e.g., obstructive sleep apnea syndrome, restless legs syndrome), being on medication for sleep, or participating in psychotherapy during the period of data collection.

Table 1 presents the general characteristics of the participants of the study, identified in the initial stage of the data collection. The age of the participants varied between 47 and 59 years old, and the diagnosis time of the FM varied between 8 and 25 years. The majority of the women (*n* = 3) reported having either elementary school (complete or incomplete), living with the spouse, and all participants met the criteria for early insomnia (difficulty to start sleep) and/or maintenance (difficulty to go back to sleep). Substantial part of the sample (*n* = 3) reported not being on health-related treatments or activities and had one of the following health problems: osteoarthrosis or spinal deviation. Two participants were in the exercise of their work activities, one was away from her duties and one unemployed; however, all continued to perform chores. All participants also had regular anti-inflammatory or analgesic use.

**Table 1:** Sociodemographic and clinical characteristics of the participants.

	P1	P2	P3	P4
**Age**	47 years old	59 years old	47 years old	58 years old
**Schooling**	Unfinished elementary school	Finished elementary school	Finished high school	Finished elementary school
**Marital status**	Common-law marriage	Married	Divorced	Married
**FM diagnostic time**	8 years	13 years	10 years	25 years
**Insomnia nature**	Beginning/maintenance	Maintenance	Beginning/maintenance	Maintenance
**Other health problems**	Osteoarthritis, column deviation	Spinal deviation hypertension	Carpal tunnel, inflate sciatic nerve, nephritis tricuspid valve tube	Synovial cyst, duodenal ulcer, osteoarthritis
**Current medications**	Anti-inflammatory	Anti-depressant, analgesic, anti-vertigo, anti-hypertensive	Anti-ulcer, analgesic	Anti-inflammatory
**Treatments/current activities**	None	None	None	Labor activity at work (20 min)
**Profession/current condition**	Access controller/unemployed	Housewife/working	Lunch lady/working	Producing assistant/working

### Material, measures, and instruments

A digital Mp4 recorder was used to record the sessions and to analyze the data, and for the evaluations we used self-report and electronic monitoring measures, namely,

Numerical pain/sleep scale-END/S (Ministry of Health, 2003): the participant was asked to indicate a grade for the intensity of her pain observed at the time of the evaluation and a grade for the quality of sleep the night before, where 0—absence of pain or impairment in sleep and 10—maximum pain or maximum loss in sleep. This measure was used continuously throughout the study, and it was also investigated, at each evaluation, whether the participant had made changes in medication, dose, and/or any type of treatment.

Fibromyalgia Impact Questionnaire, revised Brazilian version (FIQ-R) (Paiva et al., 2013): the participant indicated the symptoms from the last 7 days, among the 21 items arranged in a Likert scale (0–10), organized in three domains: (1) functionality; (2) global impact of FM; (3) intensity of symptoms.

The sum of each domain was divided by 3, 2, and 2, respectively, and from the sum of the subscores, the final score was obtained.

Short-form McGill Pain Questionnaire–revised Brazilian version (SF–MPQ) (Ferreira, de Andrade, & Teixeira, 2013): evaluated the painful experience through words (descriptors) that the participants chose to describe the pain of the evaluation. The abbreviated version consists of 15 descriptors divided into affective, sensorial, evaluative, and total dimensions. The participant should indicate that descriptors that best described the current pain in each dimension, as well as the intensity for each chosen descriptor (none, mild, moderate, severe). The sum of the points in the items resulted in the Pain Estimation Index-Total PRI, whose score ranges from 0 to 45.

Pittsburgh Sleep Quality Index, Brazilian version (PSQI-Br) (Bertolazi et al., 2011): instrument of self-report that assessed the quality of sleep in the last month, through 19 items distributed in 7 components (sleep quality, sleep latency, duration of sleep, sleep efficiency, sleep disturbances, use of sleeping medications, and daytime dysfunction), scored on a scale from 0 to 3. The sum of the components indicated the global score that varied from 0 to 21. Scores between 0 and 4 suggested good sleep quality, from 5 to 10 indicated bad sleep quality, and above 10 points, it showed the presence of sleep disturbance.

Insomnia Severity Index, Brazilian version (IGI) (Castro, 2011): instrument composed of 7 items that evaluated the severity dimensions to initiate and maintain sleep, problems to wake up in the morning, dissatisfaction with the current sleep pattern, interference of sleep problems in daytime functioning, perception of third-party sleep problems, and suffering caused by sleep difficulties. A 5-point Likert scale, where 0 = no problem and 4 = very serious problem, was used to evaluate each item, producing a total score of 0 to 28. The total score was interpreted as absence of insomnia (0–7), mild insomnia (8–14), moderate insomnia (15–21), and severe insomnia (22–28).

Actigraphy (Octagonal Basic Motionlogger®, Ambulatory monitoring, Inc. Ardsley, USA; Actwath-64®, Phillips Respironics, Inc., OR, USA): the device was placed on the wrist (of the non-dominant arm) of the participant and is intended to measure sleep quality by quantifying and analyzing the motor activity of limbs in the 24-h period. The recorded data were later transferred to a computer, and the movements were analyzed through a specialized software (Actware 6.0; Action 4) that provides objective measures of sleep, such as sleep onset latency—SL, number of awakenings—Nwak, agreed time after having started sleep—WASO, and sleep efficiency—SE. Different equipment was used due to the unavailability of single-brand appliances, and the similar performance observed in healthy individuals in the study by Tonetti, Pasquini, Fabbri, Belluzzi, and Natale (2008).

### Procedures

This study was approved by the Research Ethics Committee with Human Beings (CAAE 32623314.6.0000.5380). The participants were verbally and written explained about the ethical care and the study procedures, and those that agreed to participate in this study, signed Term of Free and Informed Consent.

The data collection was performed in a room for individual attendance of the Service-School of Psychology of a public university and in the participant’s house. The women were recruited after attending a lecture on fibromyalgia promoted by the university, among those who showed interest in the intervention and, through an interview, reported presenting the inclusion criteria established in the study. Among the women interviewed (*n* = 11), those who used sleep medications or had other sleep disorders identified by health professionals (*n* = 4) were excluded. Three women gave up participating early in the initial assessment stage, and the collection was completed with four participants.

The initial evaluations consisted of the application of the FIQ-R, SF-MPQ, PSQI-Br, IGI and the use of Actigraphy for seven consecutive days, according to standard orientation, common to all participants. To assist in the understanding of the actigraph data, participants were asked to take notes, concurrently with their use of the time they went to bed and the time they woke up. Sequentially, baseline assessments were performed using the Numerical Pain and Sleep Scale via telephone contact, on alternate days of the week and at fixed times for each participant. The baseline period lasted five alternate days (Monday, Wednesday, Friday) for participants P1 and P2, 7 days alternating for P3, and 9 days alternating for P4, or longer period if the stability criterion was not reached ( three stable average for sleep and pain, equivalent to five measures, in which the variation between averages should remain between plus or minus 1).

**Fig. 1:**
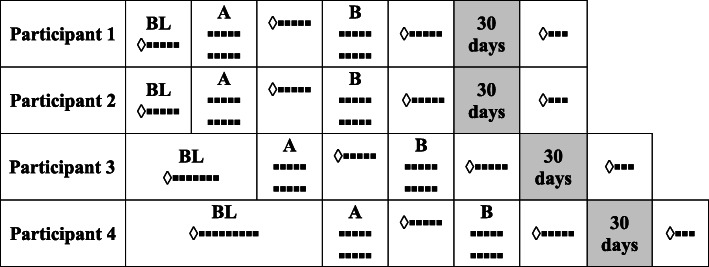
Outline with the presentation of the different phases for each participant. ◊ Initial, intermediate, final, or follow-up assessments; ▪ application of END/S; BL—baseline; A—phase A (management of conditions of the physical environment); phase B (management of interpersonal relationships). Shaded highlighting indicates the 30-day period without intervention

Intermediate, final, and follow-up evaluations were performed by applying the same instruments from the initial assessment, including the actigraphy, recording of five measures with the Numerical Pain and Sleep Scale, and three measures in the evaluation of follow-up. This study was conducted using the multiple baseline design combined with the withdrawal of intervention (Gast, 2010), and with evaluations before and after the application of each component (phases A and B). Figure 1 shows the design used with presentation of the phases for each participant.

The interventions consisted of 10 individual and in-person sessions in each phase (phase A and phase B) and weekly periodicity, corresponding to the number of sessions tested in this target population (Äsenlöf et al., 2005; Gómez-Pérez et al., 2020; Lundervold et al., 2006; Lundervold et al., 2008). The sessions were conducted by an experienced psychologist in the field. Each session lasted 1 h and 30 min, of which in the initial 20 min the Numerical Pain and Sleep Scale-END/S was applied and the activities performed throughout the week were investigated; in the next 40 min, the proposed theme for the session was worked out; and in the remaining 20 min, the training of Progressive Muscular Relaxation was performed, as instructed by Vera and Vila (2002).

The themes phase A sessions (3 to 7) were programmed according to the operant model proposed by Fordyce (1976) and Main et al. (2014), which describes the functional analysis and management of daily functional activities, and the phase B sessions (1 to 7) addressed demands of interpersonal relationships, as presented by these authors, but more specifically, followed the Social Skills Training for people with chronic pain (Penido & Rangé, 2007). An educational material was prepared to guide the application in each of the sessions of this study and it is available with the first author (for other details of the procedure, see the thesis of the first author, Kirchner, 2017, pages 123 to 125).

For phase A, the themes were (1) FM, diagnosis, and treatments; (2) cost-benefit in health care performance and effects of relaxation training; (3) identification of activities that increase pain; (4) functional analysis of environmental variables (physical conditions) related to pain response; (5) survey of positively reinforcing activities; (6) functional analysis of variables that help in coping with pain; (7) discussion about the pain maintenance cycle, (8–10) identification of progress and maintenance of therapeutic gains related to the physical environment; and for phase B, the themes were (1) pain and interpersonal relationships; (2) empathy; (3) expression of positive feelings in interpersonal relationships; (4) discrimination of feelings; (5) patterns of passive, assertive and aggressive behavior; (6) assertive communication (written activity); (7) assertive communication (role-playing); (8 to 10) identification of progress and maintenance of therapeutic gains related to the performance of socially skillful behaviors.

An analysis of two judges was conducted from the recordings of the sessions, identifying the trust between observers regarding the themes proposed for each session. A concordance calculation was performed, and the mean of 71% and 85% agreement related to the themes of the phase A and phase B sessions, respectively, were obtained.

### Data analysis

The multiple baseline design combined with the intervention withdrawal allowed to observe the effects on the dependent variables (intensity of pain and impairment in sleep quality) when introduced in each phase of intervention (independent variable), and with its withdrawal (interval between phase A and B). The instrument scores (FIQ-R, SF-MPQ, IGI, and PSQI-Br) applied at each stage were expressed as percentages, and the acrylic data were expressed by the mean and units of minutes, percentages, and quantity. The analysis of clinical significance (Jacobson & Truax, 1999) was attributed considering the general scores of the initial and final evaluations of the FIQ, IGI, and PSQI-Br instruments and the normative sample data of each instrument (e.g., standard error, standard deviation, reliability of the instrument).

The data were entered into the PsicoInfo program © 2016 version (PsicoInfo program © software online. Copyright by PsicoInfo, São Paulo, Brazil), which automatically generated the analyses and indicated if each of the participants maintained or modified the clinical status range after the intervention. Considering the availability of normative data, Jacobson and Truax (1999) suggest three criteria for analysis: criterion A is used when only normative data of the clinical population are available, criterion B when normative data are available for the non-clinical population, and criterion C when normative data are available for both populations (clinical and non-clinical).

The present study therefore used the criterion A to assess pain inability (FIQ-R) and overall pain experience assessment (SF-MPQ), criterion B to assess insomnia severity (IGI), and the criterion C to evaluate sleep quality (PSQI-Br). In the PsicoInfo program © 2016 version (PsicoInfo program © software online. Copyright by PsicoInfo, São Paulo, Brazil), the results are presented in scatter plots and descriptively, but in this study only the descriptive results were presented.

## Results

**Fig. 2:**
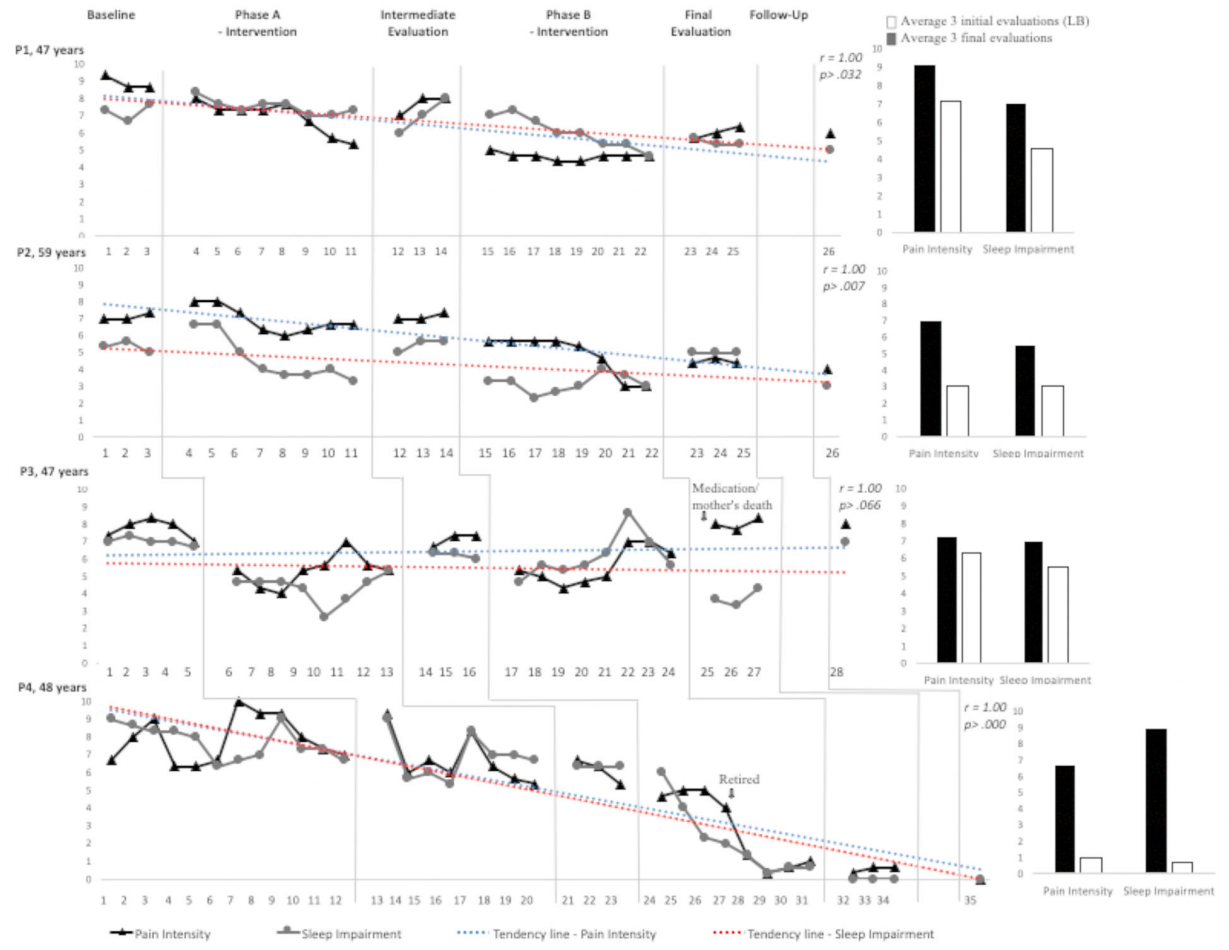
Mean of every three records of pain intensity and impairment in sleep quality (line charts). The vertical lines indicate changes in the intervention conditions. Mean of the first three and three last records of pain intensity and impairment in sleep quality (spinal charts)

To the right of the graph (Fig. 2) is the average of the first three records (beginning of the baseline) and the last three records (immediately after phase B) of each participant (*n* = 4) obtained with the Numerical Scale for Pain and Sleep. It also can be seen also shown in this figure are the line graphs between the four participants and the main changes in the routine or changes in treatments, identified as variables of potential impact on the results. The participants P1, P2, and P3 presented a decrease in the perception of pain intensity and impairment in sleep quality in intervention phase A, which increased during the intermediate evaluation week but decreased with the intervention return (phase B). Strange variables (Johnston & Pennypacker, 1993) observed at the end of the intervention (for P3) or in the middle of phase B (for P4) seem to have impacted outcomes related to pain and sleep.

While for P3 it is possible to observe a decrease in pain and sleep indications in the first sessions of phase A and B, for P4 a relatively constant decrease in pain intensity and impairment in sleep quality was observed, which in phase B occurred concomitantly with presence of weird variables (retirement).

**Fig. 3:**
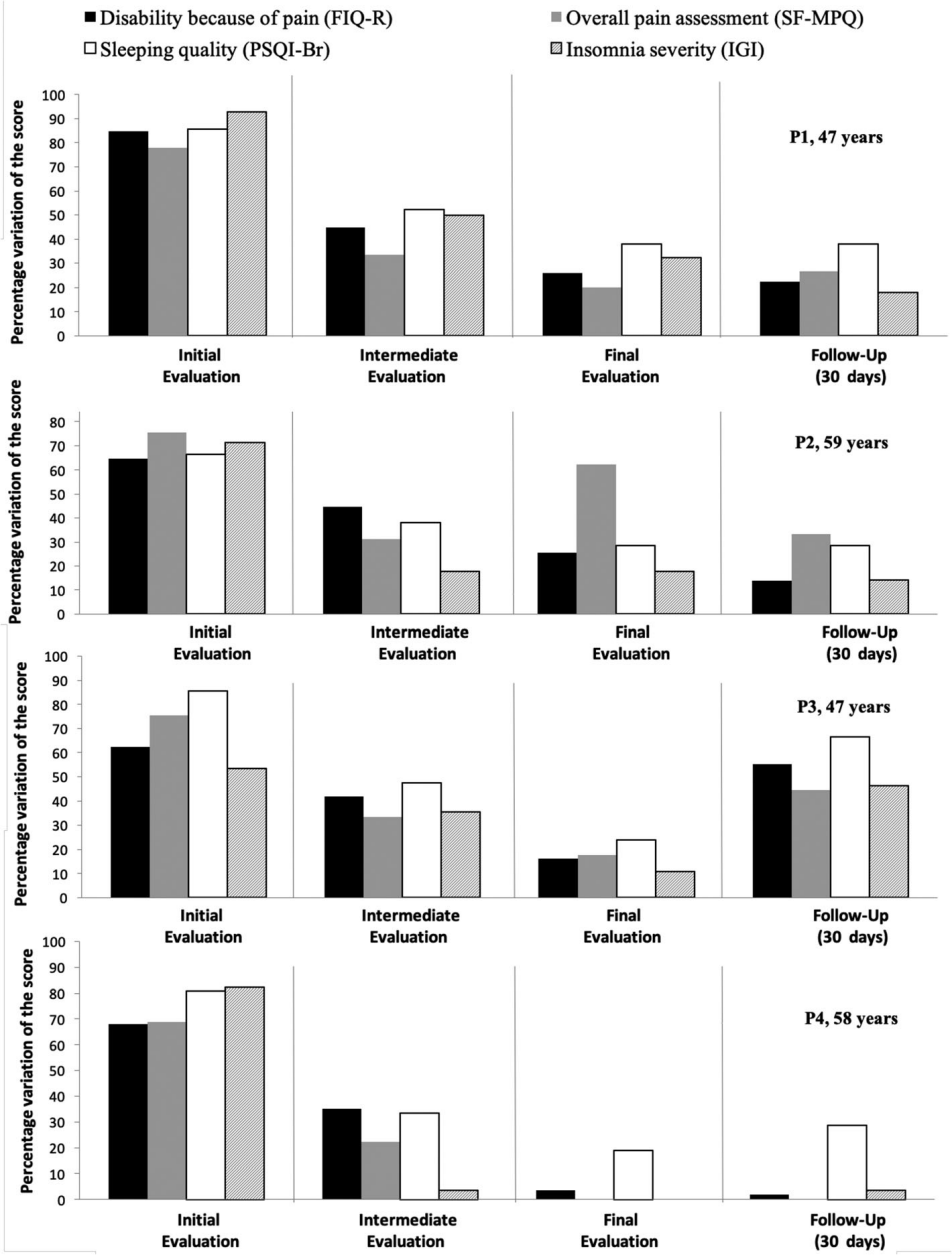
Initial, intermediate, final, and follow-up assessment data expressed in percentages for each participant

Considering the overall effect, the participants P1, P2, and P4 presented a decrease in the value of both variables at the end of the intervention, which can be observed by the trend lines and in the column charts (to the right of the figure), which presents the initial and final results (mean of the final evaluations, compared to the mean of the initial evaluations). For these participants, it is also observed that the scores assigned in the follow-up evaluation were lower compared to baseline assessments. Figure 3 shows the percentage data obtained in the initial, intermediate (after intervention phase A), final (after phase B intervention), and follow-up (after 30 days) evaluations. The FIQ-R, SF-MPQ, IGI, and PSQI-Br instruments have negative indicators, so the lower the score identified in the graph, the better the evaluated attribute.

For P1 and P3, there is a decrease in the scores of the four instruments presented in Fig. 3, after phase A and phase B intervention, with maintenance of these effects in the evaluation of follow-up (after 30 days) for P1. For P2, the scores on the instruments (FIQ-R, SF-MPQ, PSQI-Br, and IGI) were decreased during the evaluation phases, but the SF-MPQ instrument score increased after phase B, and decreased in the follow-up evaluation. Finally, P4 presented a considerable reduction of the scores for all the instruments identified in Fig. 3, which was decreasing throughout all evaluation phases, with the result of the overall pain assessment (SF-MPQ), reaching 0 in the final evaluation.

Table 2 presents the average of the records obtained by Octagonal Basic Motionlogger® and Actwath-64®. Also presented are the evaluation periods whose actigraph did not record the participant's data or those in which the participant did not use the actigraph during the night. Considering this limitation, data were analyzed only for participants 1, 2, and 3.

**Table 2:** Averages of sleep patterns recorded by actigraphy over a week

Participant evaluation	Sleep onset latency (min)	Sleep efficiency (%)	Wake time after sleep onset (min)	Number of awakenings
**P1**				
Initial	33	87	53	14
Intermediate	#	#	#	#
Final	17	97	14	5
Follow-up	▄	▄	▄	▄
**P2**				
Initial	19	96	14	8
Intermediate	15	88	17	30
Final	#	#	#	#
Follow-up	10	96	33	15
**P3**				
Initial	54	88	53	16
Intermediate	#	#	#	#
Final	30	96	23	12
Follow-up	24	82	45	19
**P4**				
Initial	53	82	33	13
Intermediate	#	#	#	#
Final	#	#	#	#
Follow-up	▄	▄	▄	▄

#The actigraph did not record the participants data

▄The participant did not use the actigraph at night

It was not possible to analyze P4 data due to absence of records after the intervention phases. Comparing the data from the initial and final evaluations (P1 and P3), for both participants, there was a reduction in latency to start sleep (difference of 16 and 24 min, respectively), and time awake during the night (difference of 39 and 30 min, respectively). Regarding the data obtained in follow-up (P2 and P3), it was verified that they were maintained for sleep onset, with a difference of 9 and 30 min, respectively, in relation to the initial evaluation.

The analysis of clinical significance generated in the PsicoInfo program © 2016 version (PsicoInfo program © software online. Copyright by PsicoInfo, São Paulo, Brazil) showed that the participant 4 went from the clinical population to the non-clinical, in all the indicators evaluated, participant 3 changed the status to non-clinical in relation to sleep quality (PSQI-Br), and participant 1 changed the status to non-clinical in relation to insomnia severity (IGI). Participant 2 remained in the clinical population for all the indicators evaluated, and none of them were moved from the nonclinical to the clinical range after the intervention.

## Discussion

This study sought to identify the effects of an analytic-behavioral intervention involving two components “handling of physical environment conditions—phase A” and “interpersonal relationship management—phase B,” on indicators of pain and sleep in women with FM and insomnia. Intermediate evaluations (return to baseline condition) were conducted in order to verify if the scores assigned in the END/S scale (dependent variables-RVs) were higher with the withdrawal of the intervention (independent variable-VI), and smaller with its introduction, attesting to one of the aspects of internal validity (Kazdin, 1982).

This effect was observed for the participants P1, P2, and P3; however, for P3, different variables identified at the end of the intervention may have affected the RVs, and it is not possible to state whether the final results of this participant were attributed to the intervention. For P4, despite the constant decrease in the scores attributed to pain intensity and impairment in sleep quality, with the score reaching 0 at the end of the study, the presence of a strange variable (retired from the work of production assistant) may have compromised the evaluation of the results throughout the study. For P1 and P2, we verified the gradual reduction in the scores of the FIQ-R, SF-MPQ, PSQI-Br, and IGI instruments, and these data correspond to those obtained with the END/S scale. There is only the caveat for P2 that the overall pain experience score (SF-MPQ) was higher at the end of the intervention, which may have occurred because of the way the instrument was applied (current pain assessment) (Ferreira et al., 2013), considering that, due to the events that occurred on the day, the participant presented severe pain at the time of the evaluation.

The actigraphy indicated an improvement in the sleep patterns for P1, especially in the reduction of the time awake during the night (difference of 39 min between the initial and final evaluation) and sleep-onset latency (16-min difference between initial and final evaluation), but it did not record the data in the final evaluation stage for P2. The data obtained for P1, grouping repeated measures and direct and self-report measurements in the pre- and post-test evaluations, attested the effectiveness of the intervention in order to meet some of the internal validity criteria pointed out by Kazdin (1982). However, this study used only self-report measures for pain assessment, and it would also be useful to adopt measures of self-registration, direct observation of behavior (Fordyce, 1976; Main et al., 2014), and electronic equipment to monitor drug intake or activity-rest rhythm throughout the day (Ancoli-Israel et al., 2003).

The results obtained with this study presents evidence that the reduction of pain can lead to improvements in sleep quality, as well as the reduction of latency to start sleep and the time spent awake throughout the night. These data are in line with other studies that point to the impact of pain on the sleep macrostructure (Finan, 2018; Keskindag & Karaaziz, 2017; Rizzi et al., 2016; Roizenblatt et al., 2001). The actigraph did not record the data in 31% of the evaluations, and the participants did not correctly use the actigraph in 12%, totalizing 47% of the lost records. Actigraphy has been a non-invasive, valid, and reliable tool for detecting sleep problems, particularly insomnia, and assessing the extent of treatment effects in the natural environment (Stone & Ancoli-Israel, 2011); however, it is not free from technical failures of the device and the non-adherence of the participants (Fekedulegn et al., 2020). These arguments point to the need to evaluate the useful life of the device, proper checking of the battery before use and registration, in addition to the need to develop strategies to promote accession. Examples of some strategies to promote adherence to the use of the actigraph: delivery of an information card about the proper use of the device and what it registers; reminders that can act as a discriminative stimulus for the use of the device; use of appliances that do not need to be removed in contact with water; presentation of the data to the participants so that difficulties in the use can be discussed.

For P3, until the final evaluation period, no different variables were found that might compromise the analysis of intervention efficacy. Until this period, the participant presented reduction of the scores attributed to pain intensity and sleep quality impairment (ND/S), especially in the initial periods of each intervention phase, and in the scores of the self-report instruments applied in the intermediate evaluation (FIQ-R, SF-MPQ, PSQI-Br e IGI). However, strange variables (death of the mother) may have affected the indicators evaluated and it cannot be said that the results obtained in the final evaluation for this participant were attributed to intervention. Due to the demands presented at the end of the study, this participant was referred for individual attendance at the psychology school service of the participating university.

The results in all self-report measures, for P4, were higher in comparison to the other participants. However, different variables may have contributed to these results. In particular, there was a considerable decrease in the scores attributed to the END/S scale when the participant retired, which may have resulted from absence of repetitive work activities, postural inadequacy, supervisor pressure on the performance of activities, among other social and occupational stressors. These data highlight the importance of modifying conditions in the physical environment (such as work activities) and managing interpersonal relationships. However, it is pointed out that, although these intervention components have been shown to be effective in improving the health conditions of people with chronic pain in general (Main et al. 2004; Morley et al., 1999), the effect of the intervention does not correspond to changes (e.g., change of job or city, death of a close family member) occurred in the life of the participants.

For half of the participants (P3 and P4), strange variables were identified that were beyond the control of the researchers. These variables are referred to as those that are supposed to impact on the dependent variable, but which do not result from experimental manipulation (Johnston & Pennypacker, 1993). The presence of different variables is a challenge in the applied research, especially when it comes to a clinical population (Taylor & Asmundson, 2008). The researcher cannot make decisions regarding the inclusion or withdrawal of medications, nor even control those stimuli that have control in the natural environment of the individual.

Therefore, it was not possible to isolate the effects of these variables, but nonetheless, the data showed evidence of effectiveness in intra-subject analysis (when the intervention is withdrawn and the change in participants’ responses is observed), and in between-subject analyzes (with the replicability of the data for the participants), and by means of the changes of score in the pre and post-test evaluations. Except for P3, the observed changes had an impact on clinical significance, shifting participants from an initially clinical status, regarding the severity of insomnia (P1 and P4), quality of sleep (P4), and disability in relation to pain (P4) for non-clinical status. According to Kazdin (1999), clinical significance can be obtained in several ways, and one of them is through the difference between the initial and final evaluations, based on the normative sample data.

One limitation of this study is that the results of clinical significance, as well as the other results, were based mainly on the participants’ reports. Further studies need to be conducted to evaluate the effect of the intervention for pain management on pain and sleep indicators in women with FM and insomnia, in order to replicate these data.

Knowledge of psychological intervention strategies that improve the quality of life and sleep patterns of individuals with FM is an area of increasing interest and practical application, which future research should continue to examine. As future implications, research could be conducted using single-case designs, enabling the isolated evaluation of the effects of each intervention component, in addition to direct measures (e.g., behavioral measures, electronic devices) along with self-report measures, in the evaluation of pain and sleep indicators.

Actigraphy is a valid and reliable measure that allows the evaluation of the extent of treatment results in the natural environment of the participant, and from their results can discuss issues of external validity. Future studies may use actigraphy not only to assess sleep patterns (sleep latency and efficiency, time awake upon at night and number of awakenings), but also as a way of measuring activity-rest rhythm during the day.

In addition, it is suggested to evaluate the impact of the intervention on the indicators of depression and anxiety, since these comorbid conditions can impact both sleep and pain in fibromyalgia.

## Conclusions

This study pointed that analytical-behavioral intervention for pain was effective in reducing problems related to sleep and pain in people with fibromyalgia. It was observed an impact on clinical significance, shifting three of the four participants from an initial clinical state to a non-clinical state. The results can be seen by two participants, and for the other two, although the changes in the scores have been significant, the presence of strange variables may have compromised the results.

Knowledge of psychological intervention strategies that improve the quality of life and sleep patterns of individuals with FM is an area of increasing interest and practical application, which future research should continue to examine. As future implications, research could be conducted using single-case designs, enabling the isolated evaluation of the effects of each intervention component, in addition to direct measures (e.g., behavioral measures, electronic devices) along with self-report measures, in the evaluation of pain and sleep indicators.

## Data Availability

We declare the datasets used and analyzed during the current study are available from the corresponding author on reasonable request.
