# Correction to: Behavior-analytic intervention for women with fibromyalgia and insomnia: a single subject design

**DOI:** 10.1186/s41155-021-00173-0

**Published:** 2021-03-11

**Authors:** Luziane de Fátima Kirchner, Maria de Jesus Dutra dos Reis

**Affiliations:** 1 Federal Univerisity of São Carlos–UFSCar, KM 235 Washington Luis, São Carlos and São Paulo, 13565-905 Brazil; 2 Dom Bosco Catholic University– UCDB, 6000 Tamandaré Avenue, Jardim Seminário, Campo Grande and Mato Grosso do Sul, 79117-900 Brazil; 3 University of São Paulo, São Paulo, Brazil

**Correction to: Psicol Refl Crít 34, 5 (2021)**

**https://doi.org/10.1186/s41155-020-00169-2**

Following the publication of the original article (de Fátima Kirchner & de Jesus Dutra dos Reis, 2021), the authors identified typesetting errors in Figs. 2 and 3. The correct figures are given below, and the original article has been corrected.

**Fig. 2:**
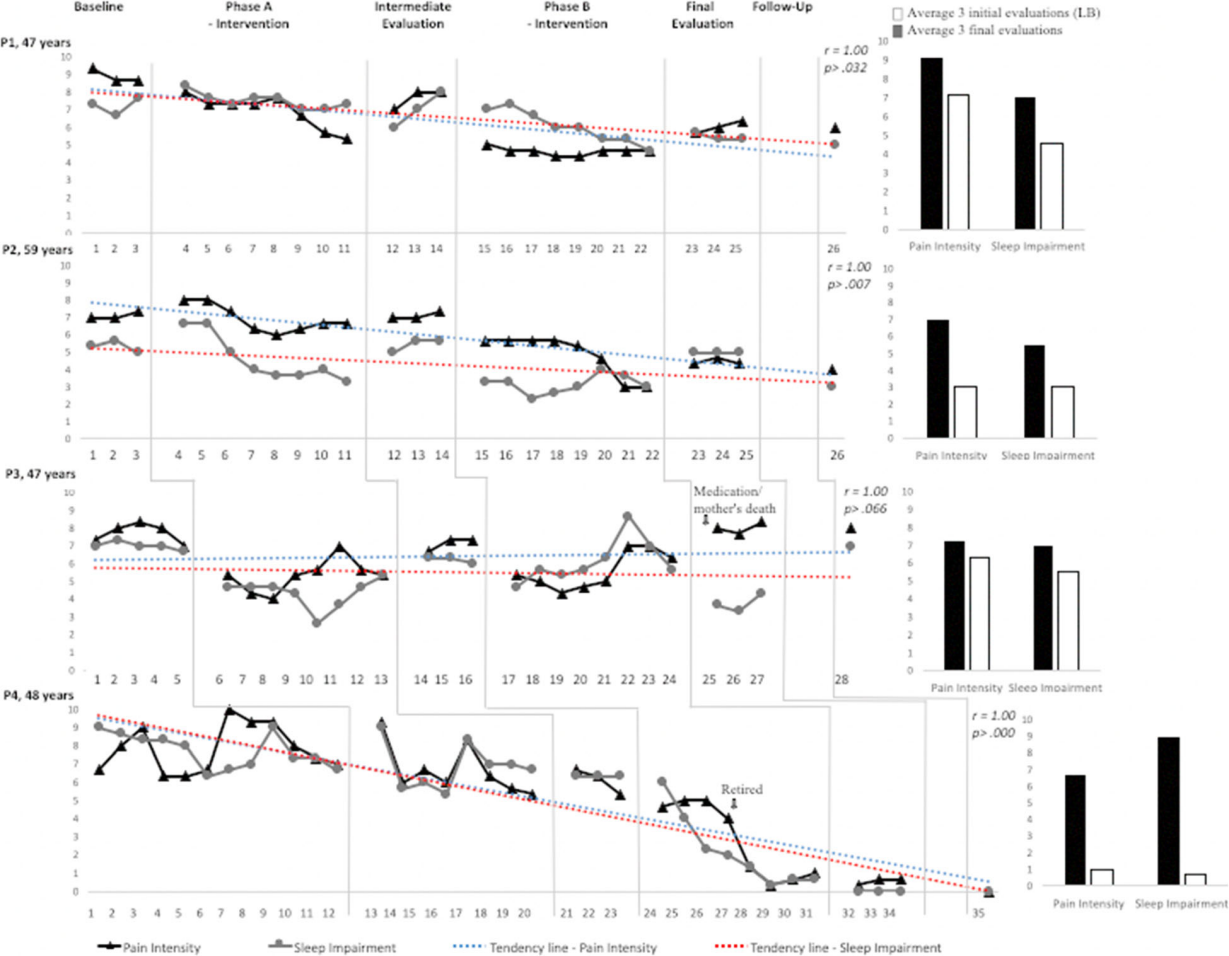
Mean of every three records of pain intensity and impairment in sleep quality (line charts). The vertical lines indicate changes in the intervention conditions. Mean of the first three and three last records of pain intensity and impairment in sleep quality (spinal charts)

**Fig. 3:**
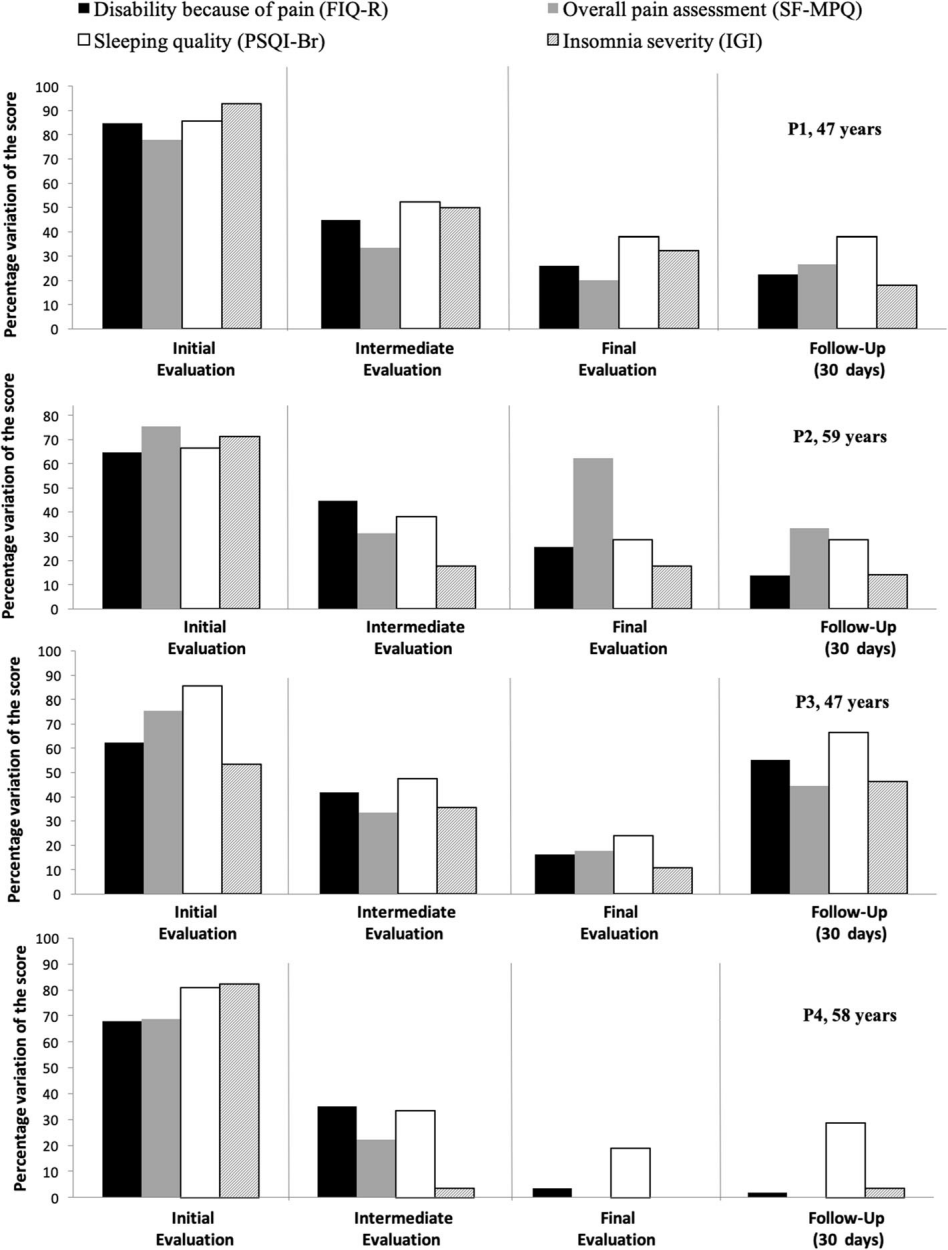
Initial, intermediate, final, and follow-up assessment data expressed in percentages for each participant
